# Mechanisms of Chemical Carcinogenesis in the Kidneys

**DOI:** 10.3390/ijms141019416

**Published:** 2013-09-25

**Authors:** Robert Radford, Helena Frain, Michael P. Ryan, Craig Slattery, Tara McMorrow

**Affiliations:** 1 UCD School of Biomolecular and Biomedical Research, Conway Institute, University College Dublin, Dublin, Dublin 4, Ireland; E-Mails: rradford@salk.edu (R.R.); helena.frain@ucdconnect.ie (H.F.); michael.p.ryan@ucd.ie (M.P.R.); craig.slattery@ucd.ie (C.S.); 2 Renal Disease Research Group, School of Biomolecular and Biomedical Science, Conway Institute of Biomolecular & Biomedical Research, University College Dublin, Dublin 4, Ireland

**Keywords:** carcinogen, kidney, proximal tubule, mechanism, bioactivation

## Abstract

Chemical carcinogens are substances which induce malignant tumours, increase their incidence or decrease the time taken for tumour formation. Often, exposure to chemical carcinogens results in tissue specific patterns of tumorigenicity. The very same anatomical, biochemical and physiological specialisations which permit the kidney to perform its vital roles in maintaining tissue homeostasis may in fact increase the risk of carcinogen exposure and contribute to the organ specific carcinogenicity observed with numerous kidney carcinogens. This review will address the numerous mechanisms which play a role in the concentration, bioactivation, and uptake of substances from both the urine and blood which significantly increase the risk of cancer in the kidney.

## 1. Cancer of the Kidney

The incidence of renal cancer in the European Union per head of population is approximately 15.8 and 7.1 per 100,000 for males and females respectively [1]. The incidence of renal cancers has declined slightly over the past decade in both the European Union and the United States following a decade-on-decade increase since 1950 [2]. The term renal cancer is an umbrella term describing a heterogeneous group of renal diseases. Multiple forms of renal cancer have been identified and they are further classified based on their location, tissue of origin and physiological properties. Renal cell carcinoma (RCC) is the most common form of renal cancer in humans [3] and is the form that will be focused on in this review

## 2. Renal Cell Carcinoma

Renal cell carcinoma (RCC) is the most prevalent neoplastic renal disease, accounting for 90%–95% of all reported renal cancers [3,4]. Clear cell renal carcinoma (ccRCC) arises from the epithelium of the proximal tubule and being the most common form of RCC, is also the most widespread form of renal cancer. ccRCC is named for the clear appearance of the cytosol when stained using common histological staining techniques due to the increased lipid accumulation.

Loss of function of the protein Von Hippel-Lindau (VHL) has been implicated in the majority of ccRCC cases. VHL in conjunction with elongin B, elongin C and cullin [5,6], form an E3 ubiquitin-ligase complex which plays a role in the ubiquitination, and subsequent proteolysis of hypoxia inducible factor-1α (HIF-1α) under normoxic conditions [7]. Hypoxia or a loss of function mutation in VHL allows accumulation and nuclear translocation of HIF-1α. Once in the nucleus, HIF-1α may homodimerise, or heterodimerise with HIF-1β whereby it functions as a co-transcription factor increasing the expression of numerous genes known to play a role in cancer including vascular endothelial growth factor, platelet derived growth factor, and the Glut-1 glucose transporter [8,9]. It is interesting to note that although the VHL mutation is observed in the majority of familial ccRCC cases, less than 4% of ccRCC cases are familial in nature, with the vast majority being accounted for by sporadic carcinogenesis (reviewed in [10,11]), yet between 60% and 70% of these sporadic cases also exhibit loss of VHL function either through point mutation, or promoter hypermethylation indicating the importance of VHL in ccRCC [12,13].

Aberrant HIF signalling not only promotes tumourigenesis through sustained mitogenic signalling and encouragement of vascularisation, but also disrupts the metabolic status of cancerous cells. As mentioned previously, Glut-1 expression is Hormone Response Element (HRE) dependent, and is commonly found to be upregulated in clear cell renal carcinoma [8]. Increased expression of Glut-1 increases glucose uptake into the cell, which facilitates the increased glucose consumption that cancerous cells are known to exhibit. Also under the transcriptional regulation of HIF are both lactate dehydrogenase (LDH) and the monocarboxylate transporter 4 (MCT4) [14,15]. Lactate dehydrogenase catalyses the conversion of pyruvate to lactate and MCT4 transports lactate out of the cell. Production of lactate by cancerous cells, and its subsequent transport to the extracellular environment helps to acidify the tumour microenvironment. This local acidification helps to further promote tumourigenesis through increased Extrcellular Matrix (ECM) degradation. It has previously been shown that in response to a decrease in pH, there is an increase in excretion of lysosomal protease cathepsin B, which helps to degrade the ECM, into the extracellular space [16]. In addition to this, it has been shown that membrane type 2 matrix metalloproteinase (MT2-MMP) is under the transcriptional regulation of HIF. Given that the ECM functions as a barrier to keep cancerous cells *in situ*, its degradation contributes towards the likelihood of a cancer spreading to areas beyond the initial lesion. In addition to this, numerous growth factors are embedded within the ECM (reviewed by [17]). Degradation of the ECM is believed to play a crucial role in tumour progression, as proteolytic cleavage of the ECM releases these latent growth factors [18].

## 3. Chemical Carcinogenicity

Chemical carcinogens are, by definition, substances which when ingested, inhaled, applied to the skin or which in other ways acquire access to tissues of the body, induce malignant tumours, increase their incidence or decrease the time taken for tumour formation [19] (Table 1). The very same anatomical, biochemical and physiological specialisations which permit the kidney to perform its vital roles in homeostasis may increase the risk of carcinogen exposure to the components of the nephron and its ancillary structures.

## 4. Susceptibility of the Kidneys to Chemical Carcinogenesis

### 4.1. Concentrating Effect

The kidneys receive approximately 25% of cardiac output and filter the plasma the equivalent volume of the entire extracellular fluid several times over each day. As a result the risk of exposure to carcinogens is disproportionately high for the epithelium of the nephron when compared to epithelial tissue found elsewhere in the body. In addition to this, one of the primary functions of the kidney is the creation of concentrated urine. Removal of waste products from systemic circulation and excretion into the urine means that as water and solutes are reabsorbed from the urine the concentration of waste products, including carcinogens increases. As such, the epithelium of the nephron would appear to be at a significantly greater risk of from blood borne carcinogens than other epithelial tissues.

### 4.2. Xenobiotic Metabolizing Capability—Bio-Activation of Pro-Carcinogens

The kidney is a biochemically active organ and contributes significantly to metabolism of xenobiotics. Lipophilic substances, for example, are not removed efficiently by the kidney and as such, addition of a polar group allows for the efficient clearance of these substances. One such class of enzymes which perform this function, also known as phase I metabolism, are the cytochrome p450 mono-oxygenases (also known as CYPs). While the majority of cytochrome p450 enzymatic activity occurs in the liver, CYPs are also expressed in other tissues including the kidney [20] and CYP activity in the kidney also contributes to the oxidative metabolism and subsequent removal of many classes of xenobiotics from systemic circulation.

While the CYP super family usually contributes to the detoxification of xenobiotics, there are instances whereby oxidation results in the creation of a toxic metabolite. The enzymatic activity of several CYP enzymes has been shown to result in the bio-activation of non-carcinogenic pro-carcinogens to their carcinogenic form. CYP1A1 for example, which is known to play a role in the bioactivation of benzo[*a*]pyrene to the carcinogenic benzo[*a*]pyrene-diol-epoxide-I-1 [21], is expressed at low levels in the kidney, however it can be induced by hydrocarbon compounds, and inter-individual differences in expression are believed to be a factor in determining the susceptibility of an individual to benzo[*a*]pyrene induced carcinogenesis [22]. NAD(P)H dehydrogenase (quinone) 1 (NQO1) is another phase I metabolising enzyme with reductive capacity. The IARC Group 1 carcinogen aristolochic acid undergoes nitroreduction to the pro-carcinogen intermediate aristolochic acid-I (AA-I) through the actions of NQO1 [23]

Phase II reactions generally involve conjugation of the phase I metabolite with glucuronic acid glutathione, amino acids or sulfonates. While conjugation usually results in the amelioration of the toxicity of a particular metabolite, there are several known instances whereby conjugation results in the bioactivation of pro-carcinogens to their carcinogenic metabolites. It has been shown that overexpression of sulfotransferase 1 (SULT1), which mediates the conjugation of a sulphate group, increases aristolochic acid DNA adduct formation [24]. Certain pro-carcinogens require a concerted effort between phase I and phase II metabolising enzymes to result in bioactivation. 2-Amino-3-methyl-9*H*-pyrido[2,3-*b*]indole (MeAαC), for example requires both CYP1A2 and SULT1A1 expression, but does not induce an increased mutation rate when either CYP1A2 or SULT1A1 are replaced with other CYP or SULT forms.

### 4.3. The Organic Ion Transport Systems

While filtration by the glomerulus is the primary mechanism for removal of xenobiotics and waste products from the blood, these compounds can also be excreted directly from the blood into the lumen of the nephron via transcellular transport. As one of the functions of the nephron is removal of xenobiotics from systemic circulation, the epithelium of the nephron is endowed with specific transport mechanisms to allow the transport of polar xenobiotics across the monolayer in such a fashion (reviewed by [25,26]).

**Figure 1:**
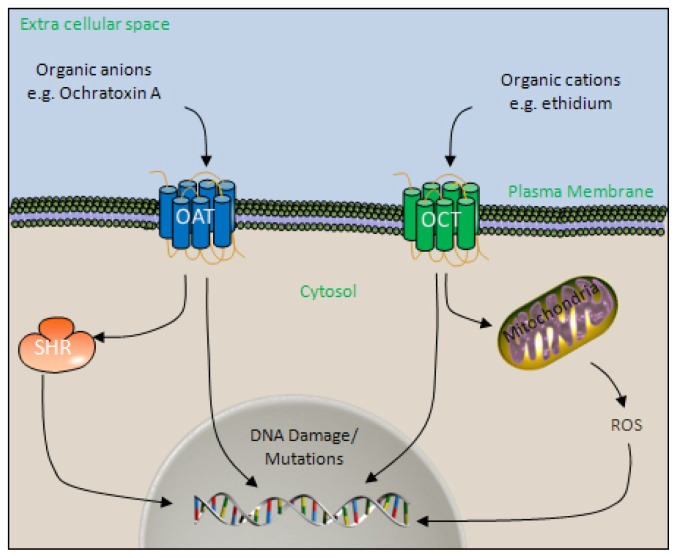
The initiation/promotion theory of cancer progression DNA damage resulting from multiple possible sources, including chemical exposure may result in the acquisition of pro-cancerous mutations (initiation). Upon detection, DNA repair mechanisms may amend the altered genome, or induce apoptosis. Failure to do so may result in the clonal expansion of the initiated cell (promotion and progression).

As this excretion pathway involves transcellular transport, anionic and cationic compounds including carcinogens, which would otherwise not cross the plasma membrane, are granted access to the cytosol and depending on the mechanism of action of the carcinogen in question may interact with the genome or interfere with other cellular processes (Figure 1).

Several carcinogens are known to be handled by the organic anion transport (OAT) family of transport proteins. Several organic anion transporters, including OAT1, OAT2 and OAT3 have been shown to have an affinity for aristolochic acid (AA), and result in a subsequent increase in uptake of the pro-carcinogen AA-I in toxicologically relevant concentrations in mouse models [27]. The organic cation transport (OCT) family has also been implicated in facilitating the uptake of potential carcinogens into the cells of the nephron. Ethidium, a mutagenic vital dye, is membrane impermeable and must be transported into the cell to gain access to the DNA. OCT1, OCT2 and OCT3 have all been shown to mediate the transport of ethidium into the proximal tubular epithelial cells of rats expressing human OCT transporters [28].

## 5. Mechanisms of Chemical Carcinogenicity

**Figure 2:**
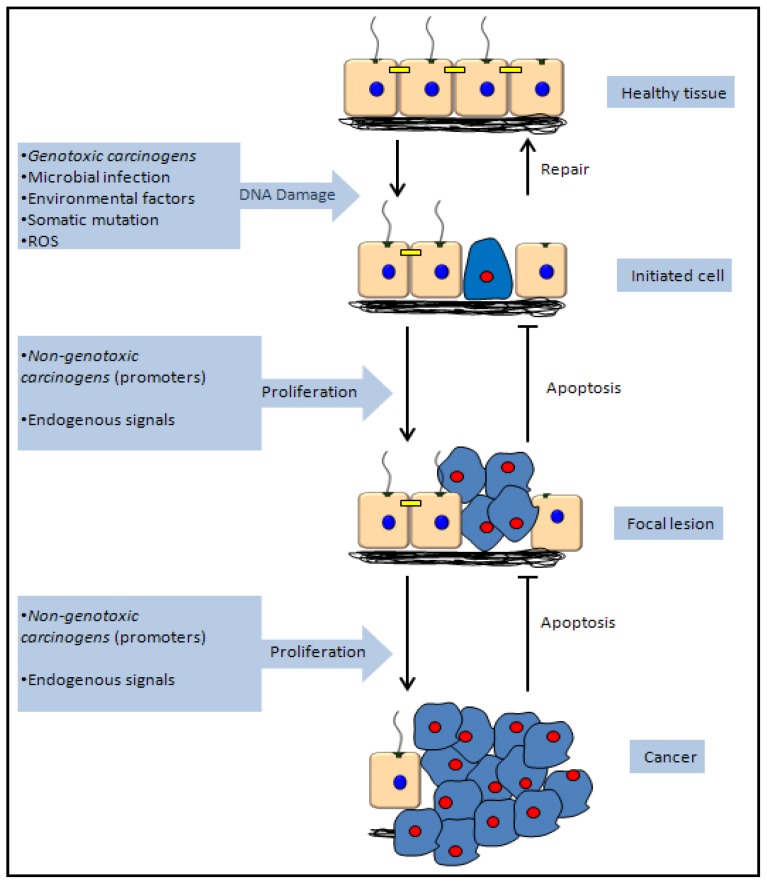
The role of the organic anion/cation transport system in carcinogenesis.

Cancer is a broad term which covers a range of distinct pathologies from solid to haematological tumours which can arise in almost any organ or tissue in the body. Given that cancer describes such a large group of related disease types it is perhaps unsurprising that there are a large number of chemical carcinogens with numerous methods of action and selectivity for various tissue types. Classification of chemical carcinogens is complex, and depending on the region, may be classified in numerous different ways. These classification methods may be simplified however, as carcinogenic agents are often simply described as either genotoxic or non-genotoxic carcinogens (Figure 2).

Polar substances cannot readily cross the plasma membrane. The transport of the polar waste products across the epithelial monolayer of the proximal tubule, is facilitated by a family of solute carrier proteins, including members of the organic anion transport (OAT) and organic cation transport (OCT) family. Polar molecules, which may not gain access to the cytosol otherwise, may do so via the actions OCT/OAT system. Carcinogens may then either directly interact with and damage DNA, in the case of genotoxic carcinogens, or may act through secondary mechanisms such as steroid hormone receptors (SHR) or reactive oxygen species (ROS) generation to promote carcinogenesis in the case of non-genotoxic carcinogens.

### 5.1. Genotoxic Carcinogenicity

Genotoxic carcinogens are agents which covalently bind, or in some other way physically interact with the DNA to induce adducts [29]. As such, genotoxic carcinogens may be regarded as initiators of carcinogenesis, capable of producing the genetic damage needed to acquire the mutations that initiate cancerous change. The majority of genotoxic carcinogens require metabolic activation. As such, in their native form, these substances are often non-carcinogenic. However, phase I metabolism results in reduction of these substances with the subsequent addition of electrophilic functional groups promoting interaction with the DNA double helix (reviewed by [30]). Covalent binding of these genotoxic metabolites leads to adduct formation, which can induce mutations in several distinct ways. Bulky adducts such as those formed by benzo[*a*]pyrene for example, may be large enough to hinder DNA polymerases, and therefore introduce mutations through the inclusion of incorrect nucleotides [31]. Numerous alkylating agents result in the formation of the N7-alkylguanine adduct which weakens the bond connecting the base of a nucleotide to the deoxyribose, thereby increasing the likelihood of an apyrimidinic or apurinic nucleotide (also known as an abasic inclusion) into the genome [31].

### 5.2. Non-Genotoxic Carcinogenicity

Non-genotoxic carcinogens are a more diverse group of chemicals in terms of their mechanisms of action and chemical properties. As opposed to genotoxic carcinogens which are capable of altering the genetic sequence and directly initiating carcinogenic mutations, non-genotoxic carcinogens may be regarded as tumour promoting agents, that is, they encourage the clonal expansion of cells which have already acquired carcinogenic mutations Non-genotoxic carcinogens do not interact directly with the DNA to promote tumour progression; however there are many physiological processes which control cell growth and division which are potential targets for these agents. Included among these are receptor and non-receptor mediated endocrine modulation [32,33], cytotoxicity and immunosuppression [34,35] and interference with gap junction intercellular communication [36]. Chloroform (Table 2d) is an example of a non genotoxic carcinogen as neither chloroform nor its metabolites are capable of interacting directly with DNA. It is able to induce low to moderate cases of renal tubule tumours in male rats, as well as damage that is localised to the proximal convoluted tubule cells in mice [37]. It is able to induce a transient increase the number of proliferating cells [38]. Gap junctions are associated with the intracellular homeostasis of growth regulatory signals and the disruption of this intracellular communication by the loss of connexin plaques (Cx) has been shown to be implicated in the cancer process. Connexins are proteins that form gap junctions. It was observed in some studies that chloroform caused a dose dependent decrease in Cx plaques containing Cx32 in proximal tubules *in vivo* [37]. However, the diversity of possible mechanisms of action of non-genotoxic carcinogens has made the identification of this class of compounds through standard carcinogen screening systems difficult. Identifying these carcinogens by classical methods becomes even more complex when it is taken into account that many of the aforementioned pathways are tissue and species specific, and that any one carcinogen may cause dysregulation of multiple pathways.

## 6. Renal Carcinogens

### 6.1. Ochratoxin-A

Ochratoxin-A (OTA) is a secondary metabolite produced by several species of the *Aspergillus* and *Penicillium genera* of moulds [39–41]. OTA contamination has been described in a wide range of foods and beverages including meat, grain, cereals, coffee, grapes, wine and beer produced globally [42–45]. OTA is a known nephrotoxin in both man and animal. In addition to these nephrotoxic effects, OTA is classed as one of the most potent rodent renal carcinogens [46,47]. Moreover, neoplastic lesions induced by OTA have been shown to be highly metastatic *in vivo* [48]. While no definitive proof exists that OTA exposure results in tumour formation in humans [49], there is sufficient evidence based on rodent testing to suggest that OTA is a possible human carcinogen and is therefore classified as a class IIb carcinogen under the International Agency for Research on Cancer (IARC) classification criteria.

Structurally, OTA is composed of a chlorinated dihydroisocoumarin bound to a phenylalanine moiety (Table 2a). As a result, OTA has been shown to compete with phenylalanine and inhibit enzymes for which phenylalanine is a substrate [50]. This is further supported by the fact that supplementation with phenylalanine has been shown to prevent OTA toxicity in an *in vivo* model [51]. Glomerular filtration of ochratoxin A is negligible as the majority is bound to plasma proteins [52], and as such, the major route of cellular entry is via the organic anion transport system, highlighting the renal specificity associated with OTA induced toxicity. *In vivo* and *in vitro* studies have shown that inhibition of organic anion transport with probenecid prevents OTA renal clearance [53,54], indicating that excretion of OTA occurs via this transcellular organic anion transport system.

However the mechanisms by which OTA induces neoplastic lesions in the proximal tubule are less clearly defined. Much work has been carried out in efforts to determine whether OTA should be classified as a genotoxic or non-genotoxic carcinogen. Given the frequency with which OTA is found as a contaminant in food and drink, reclassification of OTA as a genotoxic carcinogen would have serious implications for the food and drink industry as a 10-fold reduced human risk differential may be applied when evaluating a non-genotoxic carcinogen *versus* a carcinogen with a confirmed genotoxic mechanism of action [55]. To elucidate the mechanisms by which OTA induces genomic instability many studies have focussed on proving the existence of OTA-DNA adducts as an indicator of direct genotoxicity [56]. ^32^P-postlabelling experiments [55] in *in vitro* models have shown OTA/DNA interaction resulting in an A-2′-deoxyguanosine adduct (dGuoOTA). ^1^H-NMR studies have also shown the formation of dGuoOTA in cell-free systems [55]. The origin of such adducts has been the subject of much debate. Radiolabelling experiments thus far have failed to demonstrate the existence of direct interaction of OTA, or OTA metabolites, with genomic DNA [57–59], with several studies concluding that the proposed DNA adduct is likely a result of cytotoxicity, as opposed to genotoxicity. It has been shown that OTA exposure results in an increase in reactive oxygen species (ROS) accumulation in proximal tubular epithelial cells [60]. Abasic DNA lesions are lesions wherein hydrolysis results in the loss of a base. Such lesions result in the loss of genetic information and are therefore potentially cancer causing. Treatment with OTA has been shown to result in abasic DNA lesions *in vitro* and *in vivo* [61]. Oxidative stress resulting from the depletion of glutathione [61] and superoxide dismutase (SOD) [62] as well as increased NO [63] has been implicated in the generation of these abasic lesions.

### 6.2. Potassium Bromate

Potassium bromate (KBrO_3_) is a salt which is used as an oxidising agent. Its oxidising properties have led to it being used as a maturing agent in the processing of flour. While used in the USA as a flour maturation agent since 1914, KBrO_3_ has been banned in the E.U., Canada, Sri Lanka, China, Brazil, Nigeria and Peru as a food additive. While in the E.U. at least, KBrO_3_ is no longer a legal food additive, exposure through contaminated drinking water is still possible. The ozonation process used to sterilise drinking water, in the presence of bromine, may give rise to the formation of KBrO_3_[64]. KBrO_3_ has been shown to be nephrotoxic to humans and laboratory animals, and is currently classed as an IARC class IIb carcinogen [65].

Potassium bromate is composed of a bromic acid moiety bound to a potassium salt (Table 2b). While once commonly used as a food additive, it has been demonstrated that KBrO3 is both toxic, and potentially carcinogenic in humans. KBrO_3_ toxicity may arise as a result of the oxidative properties of the molecule. Oxidative stress has been shown to arise in numerous model systems following KBrO_3_ exposure and is believed to be the key mechanism of toxicity. It has been demonstrated *in vivo* that concentrations of malondialdehyde, a marker of lipid peroxidation and oxidative stress which are increased following KBrO_3_ exposure, are reduced when co-treated with the anti-oxidant melatonin [66]. Numerous other studies have demonstrated attenuation of KBrO_3_ toxicity in the presence of antioxidants indicating that oxidative stress is a probable causal factor in KBrO_3_ induced toxicity [67,68].

Oxidative stress is also believed to be the causal factor in KBrO_3_ induced carcinogenesis. KBrO_3_ exposure has been shown to result in the formation of the oxidative DNA adduct 8-hydroxyguanine (8-OHdG) [69,70]. Administration of numerous antioxidants, including resveratrol and vitamin E, have been shown to diminish the formation of 8-OHdG, indicating that the mutagenic activity of may be at least in part, dependent on the production of reactive oxygen species [71]. Indeed, in another *in vivo* study the antioxidant sodium ascorbic acid was shown to significantly decrease 8-OHdG adduct formation in the kidneys of both male and female mice [72].

### 6.3. Aristolochic Acid

Aristolochic acid (AA) is a naturally occurring alkaloid compound found in extracts of the *Aristolochia* species of plants which can sometimes be found in alternative medicines, mainly due to errors in plant collecting [73]. Exposure to Aristolochic acid can be unintentional or else the person can knowingly consume it in some herbal medicines that were popular in some areas of the world, in particular China, despite wide scale evidence of the damage it inflicts. AA has been singled out as the cause of Chinese herbal nephropathy (CHN) which has since become known as aristolochic acid nephropathy (AAN). AAN is a rapidly progressing fibrotic disease of the kidney, and it has been discovered that AAN patients are at a significantly higher risk of developing renal cancer [74]. Indeed AA has since been classified as an IARC Group 1 carcinogen [73], and is believed to be amongst the most potent known human carcinogens.

Aristolochic acid is composed of a mixture of related nitrophenanthrene compounds (Table 2c). The most prevalent however are 8-methoxy-6-nitro-phenanthro-(3,4-*d*)-1,3-dioxolo-5-carboxylic acid, which is commonly referred to as AAI and 6-nitro-phenanthro-(3,4-*d*)-1,3-dioxolo-5-carboxylic acid which is referred to as AAII. Both AAI and AAII are metabolised primarily by CYP1A, CYP1A2 [75] to DNA reactive species. AA-DNA adducts derived from both AAI and AAII metabolites have been identified [76,77], however the most persistent adduct in the kidney, 7-(deoxyadenosin-*N*^6^-yl) aristolactam I, known as (dA-AAI) is a known mutagenic lesion. This mutagenic lesion has been shown to give rise to transversion of adenine to thymine. Indeed several studies have shown that this transversion mutation has a particularly detrimental role in the progression of cancer. In samples from an AAN patient, the dA-AAI adduct was shown to induce mutation of AAG (lysine) to TAG (stop codon) in exon 5 of the *p53* gene [78]. Additionally, constitutive activation of H-ras has also been demonstrated in laboratory animals fed AA containing diets as a result of dA-AAI mediated A:T transversion mutations [79,80].

## 7. Screening for Chemical Carcinogens

Given the number of chemical substances that humans are exposed to each day, identifying those substances which are potential carcinogenic hazards is perhaps the most important step in the risk assessment process for chemical carcinogens. Current legislation is focused on the reduction in the volume of animal studies used for toxicological assessment (reference REACH) and as such, there is an increasing drive towards the implementation of *in vitro* models in the carcinogen screening. However, on the 1^st^ of June, 2007, new European legislation called REACH (Registration, Evaluation and Authorisation of Chemical substances) came into force. The introduction of REACH however mandates the full carcinogenicity testing of some 100,000 chemical substances. Currently, the two year rodent bioassay is the gold standard for determining the carcinogenic potential of a given substance. The use of animal studies is based on the physiological similarity that exists across mammalian species and on the assumption that agents causing cancer in animals will have similar effects in humans [85,86]. Indeed virtually all known human carcinogens have been shown to cause cancer in animal tests. While the *in vivo* rodent bioassay is the accepted “gold-standard” approach to carcinogen screening as mentioned previously, there are numerous drawbacks associated with the use of a rodent model in the implementation of the REACH legislation. The most apparent is the scale of testing required under REACH. Estimates of the number of animals necessary to fully screen the compounds covered by REACH are as high as 45 million [87], however more conservative, and perhaps more realistic estimates place the number of required animals at 12 million [88]. Regardless of the exact number of animals required, the implementation of such large scale *in vivo* screening program is clearly in contrast to the “Reduce, Refine and Replace” strategy advocated by the E.U. in terms of animal use.

## 8. Conclusions and Future Studies

The physiological functions performed by the epithelium of the proximal tubule, by their very nature, increase the risk of exposure of these cells to blood borne chemical carcinogens. The concentrating and metabolising mechanisms, which are required for the efficient removal of waste from the body, make this tissue a unique target for chemical carcinogenicity. As such, it is essential that the future of carcinogenicity testing include a renal specific component should the *in vitro* approach be adopted. Multiple E.U. projects have been implemented to develop novel, high throughput risk assessment platforms with this in mind. These projects include the EU funded ACuteTox [89,90], Predictomics [91] and carcinoGENOMICS [92], programs, which were implemented to assess the use of cell culture based organ-specific toxicity screening platforms, thereby reducing the animal burden involved in that aspect of REACH implementation. Alternative methods assessing the physiological impact of carcinogen exposure are currently being investigated in tandem [93,94], such as deciliation following carcinogen exposure [92], or colony formation using the cell transformation assay (CTA) [95,96]. The scale of these efforts highlights the gravity of the threat posed to the kidney by carcinogenic substances, and the seriousness with which these threats should be taken.

## Figures and Tables

**Table 1:** IARC carcinogen classification for compounds affecting the renal and urinary system: Several examples of chemical substances which are either known or suspected to induce cancers of the kidney and urinary system. IARC grouping: Class 1—known to cause cancer in humans, Class 2A—probably carcinogenic to humans, Class 2B—possibly carcinogenic to humans, Class 3—not found to be carcinogenic to humans.

Site	Compound	Possible source of exposure	IARC Classification
Kidney	2-Nitrofluorene	By-product of combustion	2B
	Aristolochic Acid	Use of aristolochia in herbal medicine	1
	Arsenic	Contamination of drinking water	2A
	Benzo[a]pyrene	Motor vehicle exhaust fumes	2A
	Bromodichloromethane	Chlorination of drinking water	2B
	Cadmium	Cigarette smoking, industrial activity	2A
	Chlorothalonil	Fungicide	2A
	*N*-Nitrosomorpholine	Rubber manufacturing	2B
	Ochratoxin A	Fungal contamination	2B
	Potassium Bromate	By-product of water bromination	2B
	Streptozotocin	Fungal contamination	2B

Renal pelvis and ureter	Aristolochic acid	Aristolochia species	1
	Phenacetin (and Phenacetin containing mixtures)	Non-steroidal anti-inflammatory medication	2A

Urinary bladder	2-Napthylamine	Cigarette smoking, industrial exposure	1
	4-Aminobiphenyl	Cigarette smoking, industrial exposure	1
	Arsenic	Contamination of drinking water	1
	Benzidine	Benzidine based dyes	1
	Chlornaphazine	Discontinued pharmaceutical agent	1
	Cyclophosphamide	Chemotheraputic pharmaceutical	1
	Ortho-Toluidine	Industrial/laboratory exposure	1

**Table 2:**
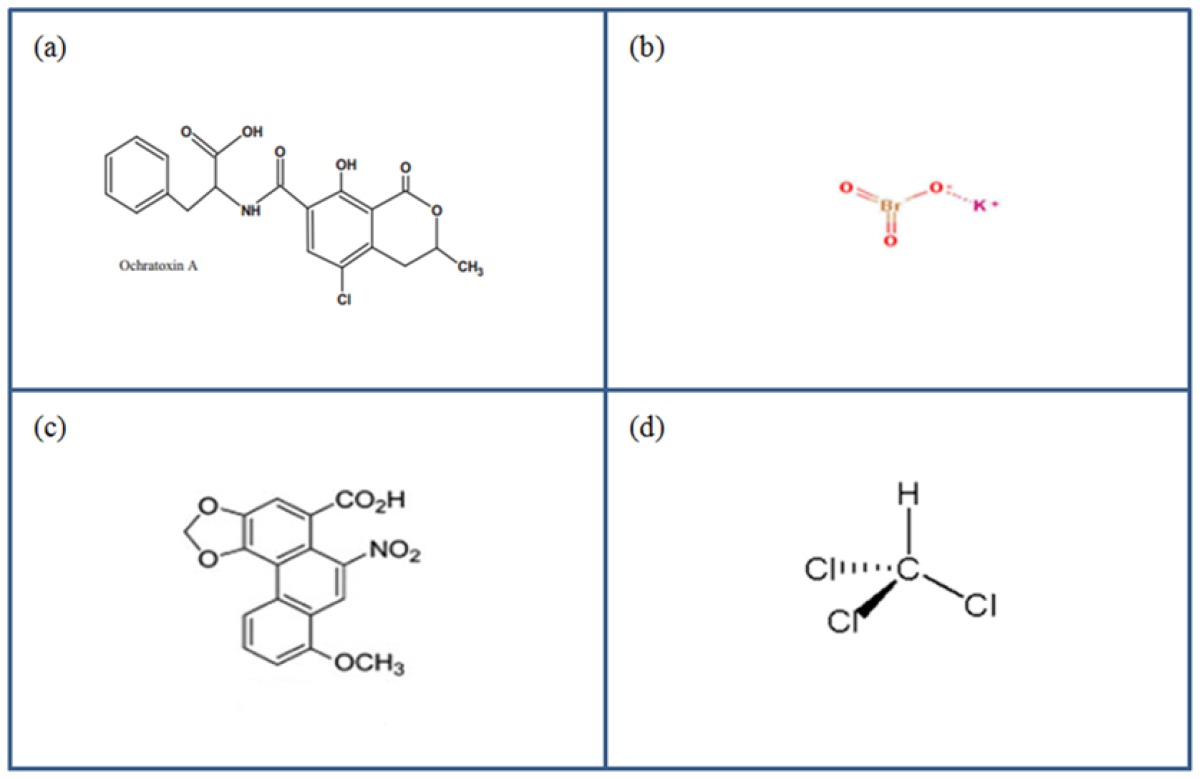
Structure of Ochrotoxin A [81] (**a**); Potassium bromated [82] (**b**); Aristolochic Acid [83] (**c**) and Chloroform [84] (**d**).
